# Guidelines for the nursing management of gestational diabetes mellitus: An integrative literature review

**DOI:** 10.1002/nop2.324

**Published:** 2019-09-30

**Authors:** Gwendolyn Patience Mensah, Wilma ten Ham‐Baloyi, Dalena (R.M.) van Rooyen, Sihaam Jardien‐Baboo

**Affiliations:** ^1^ School of Nursing and Midwifery, College of Health Sciences University of Ghana Ghana Legon; ^2^ Faculty of Health Sciences Nelson Mandela University Port Elizabeth South Africa; ^3^ Department of Nursing Science Nelson Mandela University Port Elizabeth South Africa

**Keywords:** best practices, diagnosis, gestational diabetes mellitus, guidelines, midwife, nurse, nursing management, screening

## Abstract

**Aims and objectives:**

An integrative literature review searched for, selected, appraised, extracted and synthesized data from existing available guidelines on the nursing management of gestational diabetes mellitus as no such analysis has been found.

**Background:**

Early screening, diagnosis and management of gestational diabetes mellitus are important to prevent or reduce complications during and postpregnancy for both mother and child. A variety of guidelines exists, which assist nurses and midwives in the screening, diagnosis and management of gestational diabetes mellitus.

**Design:**

An integrative literature review.

**Methods:**

The review was conducted in June 2018 following an extensive search of available guidelines according to an adaptation of the stages reported by Whittemore and Knafl (2005, *Journal of Advanced Nursing*, 52, 546). Thus, a five‐step process was used, namely formulation of the review question, literature search, critical appraisal of guidelines identified, data extraction and data analysis. All relevant guidelines were subsequently appraised for rigour and quality by two independent reviewers using the AGREE II tool. Content analysis was used analysing the extracted data.

**Results:**

Following extraction and analysis of data, two major themes were identified from eighteen (*N* = 18) guidelines. These were the need for early screening and diagnosis of gestational diabetes mellitus and for nursing management of gestational diabetes mellitus (during pregnancy, intra‐ and postpartum management). Various guidelines on the nursing management of gestational diabetes mellitus were found; however, guidelines were not always comprehensive, sometimes differed in their recommended practices and did not consider a variety of contextual barriers to the implementation of the recommendations.

**Conclusion:**

Critically, scrutiny of the guidelines is required, both in terms of the best evidence used in their development and in terms of the feasibility of implementation for its context.

**Relevance to clinical practice:**

This study provides a summary of best practices regarding the diagnosis, screening and nursing management of gestational diabetes mellitus that provide guidance for nurse–midwives on maternal and postpartum follow‐up care for women at risk or diagnosed with gestational diabetes mellitus.

## 1 INTRODUCTION

The prevalence of gestational diabetes mellitus (GDM) varies per country but is estimated to be approximately 15% among pregnant women globally (Zhu & Zhang, 2016). However, the global prevalence is expected to increase due to increasing numbers of overweight and obese women of reproductive age (Guariguata, Linnenkamp, Beagley, Whithing, & Cho, 2014; Kampmann et al., 2015). During 2003–2014, the prevalence of pregnant women with overweight and obesity increased in high middle‐income countries mainly due to increased caloric supply and urbanization and in upper middle‐ and lower middle‐income countries as a result of the decreased employment of women in agricultural activities (Chen, Xu, & Yan, 2018). GDM is defined as any degree of glucose intolerance with onset or first recognition during pregnancy (American Diabetes Association [ADA], 2010). GDM characterizes the most common metabolic complication of pregnancy and is related to maternal complications such as hypertension, pre‐eclampsia, caesarean section, infection and polyhydramnios. It is also related to foetal morbidity in terms of macrosomia, birth trauma, hypoglycaemia, hypocalcaemia, hypomagnesemia, hyperbilirubinemia, respiratory distress syndrome and polycythemia (Mitanchez, Yzydorczyk, & Simeoni, 2015; Rafiq, Hussain, Jan, & Najar, 2015).

Additionally, women diagnosed with GDM are considerably more at risk for impaired glucose tolerance and are up to six times more likely to develop type 2 diabetes 5–10 years postpregnancy compared with women with normal glucose levels in pregnancy (Work Loss Data Institute, 2016). Furthermore, children from women with GDM have a higher likelihood of developing obesity and of having impaired glucose tolerance as well as diabetes, either in childhood or in early adulthood (World Health Organization [WHO], 2016).

Some risk factors that are identified for developing GDM include age (the risk for GDM increases with age), being overweight or obese, extreme weight gain during pregnancy and a family history of diabetes. Additional risk factors related to an increased frequency of GDM include GDM during an earlier pregnancy, a history of stillbirth or giving birth to an infant with congenital abnormalities and detection of glucose in the urine as well as ethnic background (Anna, van der Ploeg, Cheung, Huxley, & Bauman, 2008; Evensen, 2012; Kampmann et al., 2015; Khan, Ali, & Khan, 2013).

Early screening and diagnosis of GDM is therefore important to prevent or reduce complications during and postpregnancy for both mother and child. Most countries use selective screening, based on the known risk factors. Although selective screening could miss GDM cases, it could also assist nursing management by focussing health resources on women with the highest risk of complications, specifically in contexts where resources are scarce. Likewise, screening early in pregnancy for pre‐existent diabetes by determining fasting glucose is justified, especially in the context of increased existence of diabetes mellitus type 2 in young women, which often remains undiagnosed (Kampmann et al., 2015).

Once women are diagnosed with GDM, management includes lifestyle modifications in terms of a diet high in dietary fibre (specifically fruit and cereal) and with a low glycaemic index, as well as routine monitoring of blood glucose levels during and postpregnancy. Additionally, if needed, the GDM is treated by means of insulin, metformin and glyburide to ensure the long‐term health of the pregnant woman and her baby (ADA, 2015; Poomalar, 2015).

A guideline, developed from rigorous evidence, would assist nurses and midwives in the screening, diagnosis and management of GDM. As they are often the first point of care for women, this is particularly important in contexts where medical care is scarce. Although some guidelines on the management of GDM exist, they are often designed for medical practitioners. No study was found that summarized best practice guidelines regarding the nursing management of GDM. This study therefore searched for, selected, appraised, extracted and synthesized data from existing available guidelines to guide the development of a best practice guideline for the nursing management of GDM.

## 2 METHODS

An integrative literature review was conducted following a five‐step process adopted from Whittemore and Knafl (2005). The processes proceeded as follows: Step 1: Formulation of the review question; Step 2: Literature searching; Step 3: Critical appraisal of evidence; Step 4: Data extraction; and Step 5: Data analysis. The integrative literature review was conducted by the first author, under supervision of the second and third authors, both of whom are experienced in conducting integrative literature reviews. The study was part of a larger study that aimed to develop a best practice guideline for the nursing management of GDM during the ante‐, intra‐ and postnatal periods.

### 2.1 Formulation of the review question

The review question (Step 1) was formulated according to the PICO format. The elements of the question were as follows: P – Population = Women; I – Issue = nursing management of GDM (including screening, diagnosis and management); C – Context = nursing and health institutions; O – Outcome = to inform best practices on the nursing management of GDM. The review question was therefore formulated as follows: What existing evidence is available to inform best practices on the nursing management of women diagnosed with GDM?

### 2.2 Literature searching process

The literature searching process (Step 2) was conducted with the assistance of an experienced librarian in selecting the databases and keywords. Inclusion and exclusion criteria were established to guide the search and selection process.

#### 2.2.1 Sources of literature

Databases were thoroughly searched using the following search engines: BioMed Central, EBSCOhost (CINAHL, ERIC, Health Source: Nursing/Academic Edition, MasterFILE Premier, MEDLINE), JSTOR, PUBMED CENTRAL, SAGE, ScienceDirect, Google Scholar, Scopus and Wiley Online Library. A manual search for guidelines was performed, using Google Scholar and Google, accessing organizations specialized in developing best practice guidelines. These included Canadian Practice Guidelines, National Guidelines Clearinghouse (NGC), National Institute for Health and Clinical Excellence (NICE), Guidelines International Network, Scottish Intercollegiate Guidelines Network (SIGN), New Zealand Guidelines Group, National Health and Medical Research Council, Registered Nurses’ Association of Ontario, American College of Obstetricians and Gynaecologists, American Diabetes Association and Health Service Executives. Grey literature, such as unpublished theses and dissertations, responding to the management of GDM were also considered.

#### 2.2.2 Key words

With the assistance of an experienced librarian, the combination of key words “guideline*” and “evidence‐based practice” and “gestational diabetes mellitus” AND “nurs* manage* OR nurs* intervention*” and “pregnan*, antenatal, intra‐natal OR postnatal*” was used. The combination of keywords used was adapted per database, if necessary, to obtain all relevant guidelines.

#### 2.2.3 Inclusion and exclusion criteria

Guidelines were included that focussed on the nursing management of GDM where any of the following aspects are addressed: early screening for GDM and its management, self‐monitoring of blood glucose levels, lifestyle modifications and/or insulin administration. Studies published in English were used as this is the language the authors are proficient in. Guidelines published between 2004–2018 were included, and the most updated version of guidelines was included. Guidelines focussing on the management of type 1 or type 2 diabetes mellitus only were excluded as were guidelines that did not consider the practices of nurses or midwives in GDM management.

#### 2.2.4 Search and selection process

**Figure 1:**
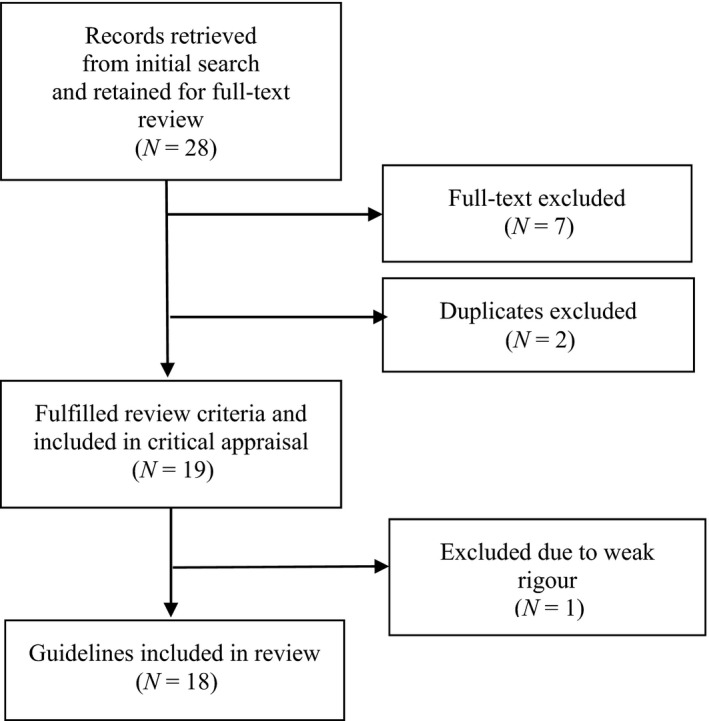
PRISMA flow of studies through the review (adapted from Moher et al., 2009)

The search for appropriate guidelines was conducted in June 2018. All guidelines that fitted the criteria for the study were retrieved and selected for inclusion. Guidelines that did not meet the required criteria were excluded. The inclusion and exclusion criteria were applied by both the first author and the fourth author (who served as an independent reviewer). Consensus regarding the inclusion and exclusion of relevant articles was reached between the authors. The search and selection process of the included guidelines is illustrated in Figure 1’s PRISMA flow chart (Moher, Liberati, Tetzlaff, Altman, & PRISMA Group, 2009).

Figure 1 shows that 28 guidelines were found in the literature search and retained for full‐text review. Seven guidelines were excluded, based on the study criteria, and two duplicates were excluded. Nineteen guidelines fulfilled the review criteria and were included for critical appraisal.

### 2.3 Critical appraisal

The AGREE II instrument was used to critically appraise the guidelines (Step 3). AGREE II consists of 23 appraisal items organized within six domains, followed by two global rating items for an overall assessment. Each domain captures a specific aspect of guideline quality. All AGREE II items were rated on a 7‐point scale (1 – “Strongly disagree”, when no relevant information was given, to 7 – “Strongly agree”, when the quality of reporting was exceptional and the criterion was fully met) (Brouwers et al., 2010). The rating for each item was done depending on the completeness and quality of reporting.

The overall score allocated to each guideline appraised was expressed as a percentage of the maximum possible score of 161. Guidelines with a score of 60 per cent were included as they were considered to have more rigour than guidelines with a lower score. Similarly, they were considered to contribute more weight to the discussion and recommendations derived from the review. Consensus was reached between the two reviewers (the first and fourth author), as a result of which one of the nineteen guidelines was excluded owing to poor rigour. A total of 18 guidelines were included for data extraction (Figure 1).

### 2.4 Data extraction process

After critical appraisal, data were extracted from eighteen guidelines (Step 4). This process was completed by the first and fourth authors, working independently. Data extraction focused on material relating to early screening and diagnosis of GDM and the nursing management of GDM.

### 2.5 Data analysis process

Thematic data analysis was used to systematically synthesize the extracted data of each guideline and develop themes (Step 5) (Burls, 2009). Consensus was achieved between the authors on the themes.

### 2.6 Ethical statement

The study obtained ethics from the University's Faculty Postgraduate Studies Committee (ethics number: H14‐HEA‐NUR‐32). The authors adhered to principles of honesty and transparency in reporting the data. Consent was not obtained, since this study had no participants.

## 3 RESULTS

Data extracted from the eighteen guidelines resulted in two main themes. They are, in outline, as follows: 1. Early screening and diagnosis of GDM; and 2. Nursing management of GDM (during pregnancy, intra‐ and postpartum management) (Table 1). Table 1 shows that most guidelines mentioned the nursing management of GDM during pregnancy (*N* = 17), followed by early screening and diagnosis of GDM (*N* = 16) and postpartum nursing management of GDM (*N* = 14). Intrapartum nursing management of GDM was least mentioned by the guidelines (*N* = 7). Table 2 provides a summary of the main recommendations per guideline, which are further discussed below.

**Table 1:** Themes per guideline

Guidelines	Early Screening and diagnosis of GDM	Nursing management of GDM			Topics covered per guideline
		During pregnancy	Intrapartum	Postpartum	
1. American Dietetics Association [ADA] (2018)		x		x	*N *= 2
2. American Association of Clinical Endocrinologists and American College of Endocrinologists [AACE/ACE] (2010)	x	x			*N *= 2
3. American College of Obstetrics and Gynaecology [ACOG] (2018a)	x	x	x	x	*N *= 4
4. Blumer et al. (2013)	x	x		x	*N *= 3
5. Diabetes Australia/Royal Australian College of General Practitioners [RACGP] (2016)	x	x		x	*N *= 3
6. CDiabetes Canada (2018)	x	x	x	x	*N *= 4
7. Diabetes Coalition of California (2012)		x			*N *= 1
8. Federation of Gynecology and Obstetrics [FIGO] (2015)	x	x	x	x	*N *= 4
9. International Diabetes Federation (2009)	x	x		x	*N *= 3
10. Kaizer Permanente (2018)	x	x	x	x	*N *= 4
11. Ministry of Health Malaysia (2017)	x	x	x	x	*N *= 4
12. National Guideline Clearinghouse [NGC] (2013)	x				*N *= 1
13. National Institute for Healthcare and Excellence [NICE] (2015)	x	x	x	x	*N *= 4
14. Queensland (2015)	x	x	x	x	*N *= 4
15. Society for Endocrinology, Metabolism, and Diabetes of South Africa [SEMDSA] (2017)	x	x	x	x	*N *= 4
16. Scottish Intercollegiate Guidelines Network [SIGN] (2017)	x	x		x	*N *= 3
17. United States Preventive Services Task Force [USPSTF] (2014)	x	x	x		*N *= 3
18. World Health Organization [WHO] (2013)	x	x		x	*N *= 3
Total no. of guidelines per phase	*N *= 16	*N *= 17	*N *= 9	*N *= 14	

**Table 2:** Main recommendations per guideline

Guidelines (*N *= 18)	ADA (2018).	AACE/ACE (2015)	ACOG (2018a)	Blumer et al. (2013)	Diabetes Australia/RACG (2016)	Diabetes Canada (2018)	Diabetes Coalition of California (2012)	FIGO (2015)	International Diabetes Federation (2009)	Kaizer Permanente (2018)	Ministry of Health Malesia (2017)	NGC (2013)	NICE (2015)	Queensland (2015)	SEMDSA (2017)	SIGN (2017)	USPSTF (2014)	WHO (2013)	Total
Early screening and diagnosis																			
Time of screening																			
First appointment/as soon as possible					x			x	x		x		x		x				*N *= 6
1st trimester														x					*N *= 1
Before 24 weeks				x						x									*N *= 2
20–24 weeks			x																*N *= 1
24–28 weeks		x		x	x	x		x		x	x		x	x			x		*N *= 10
26–28 weeks					x			x											*N *= 2
At anytime																		x	*N *= 1
Method of screening																			
50 g glucose challenge						x											x		*N *= 2
2‐hr 75 g OGTT		x		x	x	x		x	x		x	x	x	x	x	x	x	x	*N *= 14
2‐step screening test										x									*N *= 1
HbA1c								x						x					*N *= 2
Nursing management of GDM																			
During pregnancy																			
Education on GDM/glycaemic control								x					x	x					*N *= 3
Glycaemic control and monitoring		x		x	x					x			x			x	x	x	*N *= 8
Self‐monitoring	x							x	x		x		x						*N *= 5
Education self‐monitoring							x	x					x	x					*N *= 4
Support joint diabetes/antenatal specialist care													x		x		x		*N *= 3
Lifestyle moderations first line of treatment	x			x	x	x		x	x	x	x						x		*N *= 9
Insulin	x	x			x	x		x	x	x			x		x		x		*N *= 10
Metformin and glyburide		x				x		x	x		x		x		x	x	x		*N *= 9
Nutrition plan/(advise) diet	x			x	x	x	x	x	x	x	x		x	x		x	x		*N *= 13
Monitor weight gain								x			x			x					*N *= 3
Referral dietician	x				x	x		x	x				x	x					*N *= 7
Moderate exercise				x				x	x	x	x		x	x			x		*N *= 8
Education exercise			x				x						x	x					*N *= 4
Ultrasound foetal weight										x	x								*N *= 2
Test urine								x						x					*N *= 2
Nursing management of GDM ‐ Intrapartum																			
Time of delivery																			
Before 37 weeks											x								*N *= 1
Before 38 weeks											x								*N *= 1
38–39 weeks														x					*N *= 1
38–40 weeks						x				x	x		x						*N *= 4
39–40 weeks															x				*N *= 1
Before 40 weeks															x				*N *= 1
Mode of labour																			
Vaginal														x					*N *= 1
Elective (induction)						x				x	x		x						*N *= 4
Caesarean section													x	x					*N *= 2
Other recommendations																			
Close monitoring			x			x					x		x	x	x				*N *= 6
Maternal glucose level target 4−7mmol/L						x		x			x		x		x				*N *= 5
Insulin infusions						x							x		x				*N *= 3
Intravenous dextrose													x		x				*N *= 2
CSII therapy						x													*N *= 1
Cease insulin or metformin														x					*N *= 1
Nursing management of GDM ‐ Postpartum																			
Timing of blood glucose screening																			
No specified time			x													x		x	*N *= 3
24–72 hr				x										x					*N *= 2
0–6 weeks									x										*N *= 1
6 weeks											x		x		x				*N *= 3
4–12 weeks	x																		*N *= 1
6−12/13 weeks				x	x								x	x					*N *= 4
6–8 weeks to 6 months						x		x											*N *= 1
3 months										x									*N *= 1
Annual (follow‐up)										x			x		x				*N *= 3
1–3 years (follow‐up)	x								x										*N *= 2
3 years (follow‐up)			x		x	x								x					*N *= 4
Follow‐up no specified time				x															*N *= 1
Method of screening																			
75 g OGTT (using non‐pregnancy criteria)	x			x	x	x		x			x		x	x	x				*N *= 9
HbA1c					x					x			x						*N *= 3
Any test	x																		*N *= 1
Other recommendations																			
(Education on) lifestyle modifications	x		x	x	x	x		x	x		x		x	x		x			*N *= 11
Referral dietician					x														*N *= 1
Metformin	x																		*N *= 1
Discontinue blood glucose‐lowering medication immediately after delivery				x					x				x	x					*N *= 4
Breastfeeding recommended				x	x	x		x	x		x			x		x			*N *= 8

### 3.1 Early screening and diagnosis of GDM

Guidelines encourage early screening of the pregnant woman for possible identification and diagnosis of GDM, which can only be achieved if pregnant women are screened during antenatal visits. Scottish Intercollegiate Guidelines Network [SIGN] (2017) mentions a programme that must be designed for all pregnant women for early detection and treatment of GDM. Once women are screened and the results of the blood glucose tests fall within levels that can be diagnosed as GDM, the woman is considered as having GDM.

The timing of screening differs in the various guidelines. Most guidelines agree that early screening must be done at 24–28 weeks of gestation (American Association of Clinical Endocrinologists & American College of Endocrinology [AACE/ACE], 2010; Blumer et al., 2013; Diabetes Australia/Royal Australian College of General Practitioners [RACGP], 2016; Diabetes Canada, 2018; Ministry of Health Malaysia, 2017; NICE, 2015; Permanente, 2018; Queensland, 2015; International Federation of Gynaecology & Obstetrics [FIGO], 2015; United States Preventative Services Taskforce [USPSTF], 2014) (see Table 2). However, some guidelines recommend this to be done as early as possible or in the first trimester (Diabetes Australia/RACGP, 2016; International Diabetes Federation, 2009; Ministry of Health Malaysia, 2017; NICE, 2015; Society for Endocrinology, Metabolism, & Diabetes of South Africa [SEMDSA], 2017; FIGO, 2015). This often includes women that are at risk for developing GDM and, if negative, screening is repeated at 24–28 weeks of gestation (Diabetes Australia/RACGP, 2016; Ministry of Health Malaysia, 2017; NICE, 2015; Permanente, 2018; FIGO, 2015). The International Diabetes Federation (2009) specifically recommends determination of the women's risk of developing GDM at the first antenatal visit.

The method of screening recommended also differs. Most guidelines recommend the 2‐hr 75 g oral glucose tolerance test (OGTT) to aid with the diagnosis of GDM, while some guidelines opt for other tests, including the 50 g glucose challenge (Diabetes Australia/RACGP, 2016; USPSTF, 2014), the 2‐step screening test (Permanente, 2018) and the HbA1c (Queensland, 2015; FIGO, 2015). However, the AACE/ACE (2010) advises against the use of the HbA1c as a screening method to diagnose GDM, while NICE (2015) does not encourage the use of other screening tests (including fasting plasma glucose, random blood glucose, HbA1c, glucose challenge tests or urinalysis for glucose) to determine the risk of a woman developing GDM. Although the 2‐hr 75 g OGTT is recommended in most guidelines, its blood glucose values to diagnose GDM differ slightly. While some (Blumer et al., 2013; Queensland, 2015; SIGN, 2017; SEMDSA, 2017; WHO, 2013) recommend a fasting plasma glucose of 5.1–6.9 mM, 1‐hr value of >10.0 mM and 2‐hr value 8.5–11.0, according to NICE (2015), fasting values are <5.6 mM and 2 hr 7.8mM.

Specific aspects needing consideration during early screening and diagnosis are identified by various guidelines. For example, Blumer et al. (2013) recommend that the 75g OGTT be done after at least eight (8) hours night fast but not more than fourteen (14) hours. They further recommend that the usual intake of carbohydrates by the pregnant woman should not be reduced on the days preceding the OGTT test and the pregnant woman must be seated throughout the procedure. The International Diabetes Federation (2009) recommends that women that are at high risk for developing GDM should be offered healthy lifestyle advice during their first visit when screening is done. FIGO (2015) is the only guideline that considers low‐ and high‐resource contexts in their recommendations. FIGO (2015) recommends the use of a plasma‐calibrated hand‐held glucometer with properly stored test strips to measure plasma glucose in primary care settings, particularly in low‐resource countries (where a close‐by laboratory or facilities for proper storage and transport of blood samples to a distant laboratory may not exist). Using a plasma‐calibrated hand‐held glucometer may be more convenient and reliable than test results from a laboratory done on inadequately handled and transported blood samples.

### 3.2 Nursing management of GDM

Nursing management of GDM is a theme that is consistently featured in the guidelines that were included in the review. GDM management includes glycaemic control and monitoring and lifestyle modifications (diet and physical activity/exercise). Recommendations included those that should be used during pregnancy and intra‐ and postpartum.

#### 3.2.1 During pregnancy

Glycaemic control and monitoring during pregnancy must be done, for example, once a week and thereafter every 2–3 weeks until delivery (International Diabetes Federation, 2009), to keep blood glucose levels within acceptable ranges for pregnancy (AACE/ACE, 2010; Blumer et al., 2013; Diabetes Australia/RACGP, 2016; NICE, 2015; Permanente, 2018; SIGN, 2017; USPSTF, 2014; WHO, 2013). This is especially so where the woman is commenced on insulin therapy (AACE/ACE, 2010). According to Blumer et al., (2013), AACE/ACE (2010), FIGO (2015), Diabetes Australia/RACGP (2016) and ADA (2018), acceptable ranges are fasting blood sugar <5.3 mM, 1 hr pre‐prandial <7.8 mM and 2 hr postprandial <6.7 mM. Women with GDM must be encouraged to do self‐monitoring of blood glucose (ADA, 2018; International Diabetes Federation, 2009; Ministry of Health Malaysia, 2017; NICE, 2015; FIGO, 2015). FIGO (2015) recommends that self‐monitoring should be done at least daily (low‐resource settings) and up to 3–4 times a day (high‐resource settings).

As lifestyle moderations are the first line of treatment (ADA, 2018; Blumer et al., 2013; Diabetes Australia/RACGP, 2016; Diabetes Canada, 2018; International Diabetes Federation, 2009; Ministry of Health Malaysia, 2017; Permanente, 2018; FIGO, 2015; USPSTF, 2014), pharmacological treatment should only be provided if lifestyle moderations are inadequate to keep blood glucose targets within acceptable levels after 1–2 weeks (International Diabetes Federation, 2009; Blumer et al., 2013; Diabetes Australia/RACGP, 2016; Ministry of Health Malaysia, 2017; Diabetes Canada, 2018; Permanente, 2018). The preferred pharmacological treatment is insulin (AACE/ACE, 2010; ADA, 2018; International Diabetes Federation, 2009; Permanente, 2018; SEMDSA, 2017; FIGO, 2015), while metformin and glyburide can be used as effective alternatives (AACE/ACE, 2010; SIGN, 2017; FIGO, 2015) if not contraindicated or unacceptable for the woman (NICE, 2015). However, metformin should be prescribed/continued under specialist supervision (SEMDSA, 2017) but is not approved in Australia (Diabetes Australia/RACGP, 2016).

Health education should be provided on GDM and glycaemic control, especially on recognizing the signs of hypoglycaemia and treatment of those signs. Women should be made aware of the implications of GDM for the woman and the foetus and of steps to achieve management of GDM. Family members should be taught how to use the glucometer, as well as the management principles and importance of long‐term follow‐up (Diabetes Coalition of California, 2012; NICE, 2015; Queensland, 2015; FIGO, 2015).

In terms of diet, it is recommended that pregnant women with GDM receive nutrition counselling (Blumer et al., 2013; Diabetes Australia/RACGP, 2016; NICE, 2015; SIGN, 2017; USPSTF, 2014), preferably from a dietician familiar with GDM (ADA, 2018; Diabetes Canada, 2018; NICE, 2015; Queensland, 2015; FIGO, 2015). The nurse or midwife must make it a point to involve all the necessary healthcare professionals (Queensland, 2015) and preferably those with expertise in GDM (International Diabetes Federation, 2009; SEMDSA, 2017). A healthy diet should be high in vegetables and protein (Permanente, 2018) and low in GI (International Diabetes Federation, 2009; NICE, 2015). The recommended diet should consist of a minimum intake of 1,600–1,800 kcal/day and carbohydrate intake limited to 35%–45% of total calories (Blumer et al., 2013; Ministry of Health Malaysia, 2017; Diabetes Canada, 2018). Weight gain in the pregnant woman with GDM must also be checked according to her BMI (Ministry of Health Malaysia, 2017; Queensland, 2015; FIGO, 2015). The nurse or midwife must encourage the pregnant woman with GDM to stick to the diet or nutrition planned with the dietician and also to monitor her blood glucose levels as scheduled.

In terms of exercise, moderate exercise is recommended, such as a 30 min’ (at least 10‐min periods) (Queensland, 2015) walk after meals (Blumer et al., 2013; NICE, 2015) or 1 hr a day (Permanente, 2018). Education should also be given about armchair exercises (American College of Obstetrics & Gynaecology [ACOG], 2018a).

To provide the best nursing management for GDM, a customized plan of care, especially for women at high risk, should be developed (NICE, 2015) that is individualized and culturally sensitive (International Diabetes Federation, 2009). This care plan could also include checks of blood pressure and dipstick urine protein every 1–2 weeks (resourced settings) or monthly (low‐resource settings; FIGO, 2015; International Diabetes Federation, 2009; Queensland, 2015) as well as an ultrasound between 30–32 weeks of gestation to estimate foetal weight (Queensland, 2015) or every four weeks from 28–36 weeks of gestation (Ministry of Health Malaysia, 2017).

#### 3.2.2 Intrapartum

Although guidelines differ regarding the delivery time and mode, most agree with an elective induction of 38–40 weeks to reduce the risk for stillbirths (Diabetes Canada, 2018; Ministry of Health Malaysia, 2017; NICE, 2015; Permanente, 2018). A caesarean section around 40 weeks plus 6 days is recommended, but this should be done before that time for those with comorbidities or maternal or foetal complications (NICE, 2015; Queensland, 2015). The primary objective of the intrapartum nursing management of GDM is to maintain maternal euglycemia to prevent neonatal hypoglycaemia, which is caused by the hyperinsulinemia in the baby due to hyperglycaemia in the mother. Close monitoring of women with GDM during labour and delivery should therefore be done (ACOG, 2018a; Diabetes Canada, 2018; Ministry of Health Malaysia, 2017; NICE, 2015; Queensland, 2015; SEMDSA, 2017) at least once an hour (ACOG, 2018a) or, according to NICE (2015), every thirty (30) minutes till delivery. Maternal blood glucose levels must be maintained between 4.0 mM–7.0 mM (Diabetes Canada, 2018; Diabetes Coalition of California, 2012; Ministry of Health Malaysia, 2017; NICE, 2015; SEMDSA, 2017). To achieve these blood glucose levels, the woman should be given enough glucose during labour to help her to cope with the high level of energy demands for labour and for delivery so as to prevent the woman from having hypoglycaemia (Diabetes Canada, 2018; NICE, 2015; SEMDSA, 2017). NICE (2015) recommends that, if the capillary plasma glucose is above 7 mM, intravenous dextrose and insulin infusion must be given during labour and delivery, although the guideline does not specify how much.

#### 3.2.3 Postpartum

Postpartum nursing management of GDM constitutes a critical challenge when treating women with GDM. Various guidelines selected for synthesis focus on postpartum management. It is recommended blood glucose‐lowering medication should be lowered immediately after delivery (International Diabetes Federation, 2009; Blumer et al., 2013; FIGO, 2015; Queensland, 2015; Diabetes Australia/RACGP, 2016; Ministry of Health Malaysia, 2017; Diabetes Canada, 2018). Although guidelines recommend postpartum blood glucose screening for early detection of diabetes mellitus, impaired glucose tolerance or impaired fasting glucose (ACOG, 2018a), they differ on when this should be done. Most guidelines recommend 6 weeks when the woman comes for postnatal follow‐up (Ministry of Health Malaysia, 2017; NICE, 2015; SEMDSA, 2017) or between 6–12/13 weeks (Blumer et al., 2013; Diabetes Australia/RACGP, 2016; NICE, 2015; Queensland, 2015). Blumer et al. (2013) is the only guideline that recommends, besides the 6‐ to 12‐week screening, that blood glucose monitoring should also be done 24–72 hr after delivery. This is to rule out high blood glucose levels just after delivery.

Most guidelines prefer a follow‐up of screening varying between 1 year (NICE, 2015; Permanente, 2018; SEMDSA, 2017) and 3 years (ACOG, 2018a; Diabetes Australia/RACGP, 2016; Diabetes Canada, 2018). According to ADA (2018), risk factors should be considered when deciding the timeframe for follow‐up screening. According to Diabetes Canada (2018), emails and phone calls can be used to remind women for their follow‐up screening. The method of screening recommended also differs, although a 2‐hr 75 g OCTT seems to be the most frequently used, as recommended by nine (*N* = 9) guidelines. ACOG (2018a) recommend that women with impaired glucose tolerance or with impaired fasting glucose must be referred as early as practicable for prevention therapy.

In addition, women with a history of GDM must be counselled on preventative lifestyle modifications to reduce the risk of type 2 diabetes (ACOG, 2018a; ADA, 2018; Blumer et al., 2013; Diabetes Australia/RACGP, 2016; Diabetes Canada, 2018; NICE, 2015; Queensland, 2015; SIGN, 2017; FIGO, 2015) specifically regarding their diet, weight control and exercise requirements (SIGN, 2017). Referral to a dietician can be done (Diabetes Canada, 2018). According to NICE (2015) women should be educated specifically with regard to the signs and symptoms of hyperglycaemia. Education on the risk of developing GDM in subsequent pregnancies should be included as well as the benefits of optimizing postpartum and inter‐pregnancy weight (Queensland, 2015).

Various guidelines (American College of Obstetrics and Gynaecology [ACOG], 2018b; International Diabetes Federation, 2009; Blumer et al., 2013; FIGO, 2015; Queensland, 2015; Diabetes Australia/RACGP, 2016; Ministry of Health Malaysia, 2017; Diabetes Canada, 2018) recommend that women with GDM should be encouraged to breastfeed their newborns immediately after delivery, thereby helping to prevent hypoglycaemia in the newborn. It is recommended that continuous breastfeeding should be done for at least 3–4 months postpartum (Diabetes Canada, 2018; SIGN, 2017) or longer (Ministry of Health Malaysia, 2017) as this helps to reduce childhood obesity, glucose intolerance and diabetes later in life. However, caution should be advised regarding maternal hypoglycaemia if breastfeeding (SEMDSA, 2017) and skilled lactation support is therefore recommended (Queensland, 2015; FIGO, 2015). Finally, extra attention is also required to detect early signs of genitourinary, uterine and surgical site infections (in the case of an episiotomy and caesarean delivery; FIGO, 2015).

## 4 DISCUSSION

### 4.1 Comprehensiveness of the guidelines

Several guidelines from a variety of healthcare organizations, associations or health departments were found that include aspects relevant to the nursing management of GDM. Not all guidelines focus on all aspects (namely glycaemic control, monitoring and treatment and lifestyle moderations, including diet and physical activity/exercise) and phases of the nursing management of GDM (during pregnancy, intrapartum as well as postpartum) as only 8 (*N* = 8) of the guidelines reviewed include all phases of the management of GDM (ACOG, 2018a; Diabetes Canada, 2018; NICE, 2015; Permanente, 2018; Queensland, 2015; SEMDSA, 2017; FIGO, 2015). There were guidelines which cover some of the phases or the nursing management of GDM in general. For example, the SIGN (2017) guideline does not focus on the nursing management of GDM during labour and delivery but does provide general recommendations on what should be done during pregnancy and postdelivery. NGC (2013) also does not discuss intrapartum nursing management of GDM but gives recommendations on the testing and diagnosis of pregnant women.

Guidelines also differed in the level of descriptiveness employed. Guidelines that were generally more descriptive with their recommendations included those from Blumer et al. (2013), AACE/ACE (2010), FIGO (2015), NICE (2015), SEMDSA (2017) and Diabetes Canada (2018). Additionally, variances in best practices regarding screening and diagnosis as well as the nursing management of GDM were observed. It is thus recommended that existing guidelines should be scrutinized in respect of their level of descriptiveness, together with the latest best evidence and of the quality of the evidence used to develop the recommendations in the guidelines.

### 4.2 Quality of evidence

Not all guidelines reviewed included the level or grades of evidence used for each recommendation and various levels or grades were used. This is required to select a recommendation for implementation that fits the context best and will yield the best outcomes for both mother and child. For example, some of the guidelines included did not use a grading system for evidence or references when citing the recommendations (Diabetes Coalition of California, 2012; NGC, 2013; NICE, 2015; Permanente, 2018), while others did not use a grading system for the evidence included, but did use a variety of evidence when citing the recommendations (International Diabetes Federation, 2009; Queensland, 2015; SEMDSA, 2017; USPSTF, 2014). Other guidelines included grading systems for the evidence of which an A–D grading system was the most commonly used which was adapted from the American Diabetes Association (2018). Grade A refers to clear evidence from well‐conducted, generalizable RCTs, grade B includes supportive evidence from well‐conducted cohort studies, while grade C and grade D refers to supportive evidence from poorly controlled or uncontrolled studies as well as expert consensus or clinical experience, respectively. Some guidelines included a variety of evidence supporting the recommendations (grade A–D) (AACE/ACE, 2010; Diabetes Australia/RACG, 2016; WHO, 2013), with two guidelines mainly using grade A and B evidence (ADA, 2018; Blumer et al., 2013), another two guidelines mainly using grade B and C evidence (ACOG, 2018a; SIGN, 2017) and a fifth guideline mainly using grade C and D evidence to support the recommendations (Diabetes Canada, 2018). FIGO (2015) used the 2019 grading system, including mostly moderate quality evidence (+++) and very low‐quality evidence (+), while the guideline by the Ministry of Health Malesia (2017) used a grading system from the United States/Canadian Preventive Services Task Force (2001) where level I (at least one properly conducted RCT) and level III (expert opinions) were mostly used to support the recommendations. Therefore, in this review it was impossible to make a valid statement for each recommendation that was based on evidence grades/levels. A systematic review is therefore recommended which extends beyond the AGREEII tool that was undertaken in this study to summarize the overall strength of evidence of each recommendation, such as the screening, diagnosis and nursing management of GDM during pregnancy, intrapartum and postpartum care and the overall quality of each particular guideline. Additionally, only two guidelines considered the input from the woman in the management of GDM (International Diabetes Federation, 2009; NICE, 2015). Any recommendation or care plan developed should be discussed with the woman diagnosed with GDM and her permission should be obtained to implement the recommended care practices.

### 4.3 Resources/Barriers

Only one guideline considered the context in terms of low/high resources (FIGO, 2015). The reality is that most low‐resource countries are unable to implement some of the recommendations, such as, for example, universal 75‐g OGTT or self‐monitoring every day (FIGO, 2015). The possible barriers to the implementation of the recommendations caused by a lack of resources were not addressed in most of the guidelines. For example, several barriers to maternal health related to GDM have been identified. These include the lack of trained healthcare professionals; high staff turnover; lack of standard protocols and diagnostic tools, consumables and equipment; inadequate levels of financing of health services and treatment; and lack of or poor referral systems, feedback mechanisms and follow‐up systems.

Further barriers relate to distance to health facility; perceptions of female body size and weight gain/loss related to pregnancy; practices related to a pregnant women's diet; societal negligence of women's health; lack of decision‐making power among women regarding their own health; the role of women in society and expectations that the pregnant woman move to her maternal home for delivery; and lack of adherence to recommended postpartum screening and low continued lifestyle modifications (2017, & Stray‐Pederson, 2^2017^; Nielsen, Courten, & Kapur, 2012; Nielsen, Kapur, Damm, Courten, & Bygbjerg, 2014). Additionally, a recent delivery experience, baby's health issues, personal and family adjustment to the new baby, a negative experience of medical care and services and concerns about postpartum and future health (as in, for example, fear of being informed that they have diabetes) were specifically related to the barriers to postpartum follow‐up care (Bennett et al., 2011).

The barriers cited should be considered when implementing the recommendations offered by the guidelines. Further, an integration of health services should be offered as well as communication between the different healthcare professionals is required. Integration of health services can be done when postpartum follow‐up of a mother can be combined with the child's vaccination and routine paediatric care.

### 4.4 Recommendations

Kaiser and Razurel (2013) examined the determinants of health behaviours during the postpartum period in GDM patients. They found that the women's physical activity and diet do not often meet the recommended health‐promoting actions. Risk perception, health beliefs, social support and self‐efficacy were the main factors that were identified as having an impact on the adoption of health behaviours. GDM clients are encouraged to engage in lifestyle modifications or healthy behaviours during the postpartum period. It is important, therefore, to identify the factors that may influence these clients to continue with healthy behaviours (Kaiser & Razurel, 2013).

Education of the woman diagnosed with GDM on the screening, and management (including preventative lifestyles) is imperative and will assist in addressing some of the above‐mentioned barriers. Education, as mentioned by most guidelines, should preferably be given by nurses and/or midwives to all pregnant women that are at risk or diagnosed with GDM. Furthermore, the healthcare professionals will need to be trained on pregnancy‐specific lifestyle modifications, treatment and screening for complications (International Diabetes Federation, 2009). Finally, it is particularly important for low‐resource settings that availability of trained healthcare professions, self‐monitoring equipment and insulin supply, and laboratory resources for clinical monitoring of glucose control and assessment of renal damage (International Diabetes Federation, 2009) should be prioritized in national budgets for health care.

No contextualized guideline on the nursing management of GDM is available for contexts where women with GDM deal with specific challenges such as factors related to the health system, or socioeconomic and cultural conditions that may impose barriers to the implementation of the best practice. It is therefore recommended that, prior to the implementation, a context analysis should be conducted to identify specific barriers to its implementation. This was confirmed by FIGO (2015) who mentioned that local decisions will be required to decide whether a selective or universal approach will be used for each individual patient. Additionally, further research of the barriers is required to develop contextualized guidelines considering the challenges some women and some health systems may have in accessing or providing adequate maternal health care. The developed contextualized guidelines could then be piloted. Piloting will be done to determine how the guidelines could have a positive effect on the nursing management of GDM while considering the input from the pregnant women as well as possible barriers or resource constraints towards its implementation.

### 4.5 Limitations

Some limitations of the study were observed. A comprehensive search of a variety of databases available to the authors was used with the assistance of an experienced librarian. However, limited databases were available, and some organizations/ developers of guidelines were not subscribed to so some guidelines may have been missed. Although the reviewer possessed wide experience in appraising the guidelines, more independent reviewers could have reduced possible bias in the selection process of the guidelines.

## 5 CONCLUSION

Data extracted from the eighteen guidelines resulted in two main themes: 1. Early screening and diagnosis of GDM; and 2. Nursing management of GDM (during pregnancy, intra‐ and postpartum management). Although a variety of guidelines on the management of GDM were found, guidelines were not always comprehensive, sometimes differed in recommended practices and did not consider barriers to the implementation of the recommendations.

## 6 RELEVANCE TO CLINICAL PRACTICE

This study provides a summary of best practices regarding the diagnosis, screening and nursing management of GDM. The findings can be used by nurse–midwives when conducting maternal and postpartum follow‐up care for women at risk or diagnosed with GDM. However, critically scrutinizing the guidelines in terms of the best evidence used in their development and feasibility of the implementation of the recommendations for its context is required. Additionally, education of women with GDM could assist in addressing any barriers such as certain harmful health beliefs, a lack of social support and self‐efficacy to provide the best maternal health care. Further research is recommended to determine the strength of evidence of each recommendation and the development and implementation of a contextual guideline on the management of GDM that considers possible barriers and resource constraints towards its implementation.

## CONFLICT OF INTEREST

The authors have no conflicts of interest to disclose.
